# Study on the medical behaviors of residents in Yicheng District of Zhumadian

**DOI:** 10.3389/fpubh.2025.1610825

**Published:** 2026-01-06

**Authors:** Ye Nie, Ying Du

**Affiliations:** Hunan University of Chinese Medicine, School of Humanities and Management, Changsha, China

**Keywords:** healthcare-seeking behavior, Anderson’s healthcare utilization model, hierarchical diagnosis and treatment, influencing factors, residents

## Abstract

**Introduction:**

The current healthcare system layout is not adequate, with insufficient high-quality medical resources and unreasonable allocation, leading people to prefer large hospitals regardless of the severity of their illness, while fewer individuals seek medical care at the primary medical institutions. The pattern of hierarchical diagnosis and treatment has not yet been formed.

**Methods:**

This study examines the healthcare-seeking behavior of residents in Yicheng District, Zhumadian City, and analyzes the differences in their healthcare-seeking behavior, discussing the problems associated with their choices. This study is primarily based on Anderson’s healthcare utilization model and behavioral theory to design the questionnaire, utilizing multiple independent samples rank sum tests and multiple comparisons for analysis.

**Results:**

The findings are as follows: (1) Residents exhibit a strong tendency toward self-diagnosis; (2) Residents tend to choose higher-level institutions over lower-level ones and familiar institutions over unfamiliar ones when seeking medical care; (3) Primary medical institutions lack adequate service capabilities.

**Discussion:**

In this regard, the following countermeasures are proposed: (1) Enhance residents’ medical and health literacy and guide them to seek medical care in an orderly manner; (2) Improve the service level of primary medical institutions; (3) Strengthen the healthcare security mechanism at the primary level.

## 1 Introduction

The current social and economic transformations are leading to increasing basic health needs among the population. This places greater demands on the medical and health service system. Nonetheless, the irrational allocation of medical and health resources presents a significant obstacle to the development of China’s medical and health services. The World Health Organization holds the belief that primary health care locations can solve 80% of health problems. Meanwhile, in China, 80% of medical resources are concentrated in urban areas, with the other 80% further concentrated in large and medium-sized hospitals. This process leads to the formation of an inverted pyramid structure. There is a long-standing belief that large hospitals employ superior medical technology compared to other health care institutions (1). Large hospitals consequently experience a patient overload. Meanwhile, many primary health care facilities are underutilized and treat far fewer patients than expected. In 2015, the General Office of the State Council issued “Guiding Opinions on Promoting the Construction of a Hierarchical Diagnosis and Treatment System.” This policy aimed to promote the rational distribution of medical and health resources and to guide residents in receiving orderly and reasonable medical treatment. Zhumadian City responded actively to the national policy. The city focused on promoting the construction of the hierarchical diagnosis and treatment system and improving the medical and health service system. The office set up 24 close-knit county medical communities in nine counties, covering 222 medical institutions. It also pushed forward reforms of the medical insurance payment system, such as medical insurance prepayment, payment by disease type, and Disease Diagnosis-Related Grouping. During the implementation of the “Outline of the Thirteenth Five-Year Plan for the National Economic and Social Development of the People’s Republic of China,” the actual outpatient medical insurance reimbursement rate in the city increased from 57 to 68.2%. The actual reimbursement rate for inpatient hospitalization costs increased from 61.8 to 63.9%. However, significant differences exist in medical standards, human resources, management, service quality, and economic levels among medical institutions at all levels. Such variation explains why patients prefer hospitals over primary medical institutions for treatment. Patients believe primary medical institutions lack advanced medical equipment and supplies, which makes it difficult to gain their trust (2).

Residents’ medical behaviors are complex and influenced by various psychological and social factors, which can lead to different outcomes (3). This paper investigates the medical behavior of residents in Yicheng District, Zhumadian City, Henan Province; explores whether there is a significant difference in the choice of medical behavior of residents in Yicheng District; analyzes the reasons for residents’ unreasonable medical treatment; and puts forward corresponding countermeasures to rationally allocate medical and health resources, guide residents to seek medical treatment reasonably, promote the system of hierarchical diagnosis and treatment, and better satisfy residents’ increasing demand for health services.

## 2 Relevant concepts and theoretical foundations

Most scholars define health care accessibility in terms of spatial factors. Health-seeking behavior is a broad concept, and there is currently no universally accepted definition within the academic community. Research on health-seeking behavior first emerged in the field of Western medical sociology. According to Kasl, health care behavior is the act of an individual who actively seeks medical care after recognizing the existence of his disease and trying to reduce the pain that it causes. Choosing an appropriate disease treatment, the kind of medical institution to receive treatment, and other aspects are included in this process (4). “The process by which individuals respond to physical symptoms in various ways, monitor their internal condition, identify and interpret bodily symptoms, determine the causes of illness, take therapeutic measures, and utilize formal and informal healthcare resources” is the way McKenzie defined healthcare-seeking behavior. The definition of healthcare-seeking behavior he proposed not only includes seeking medical advice but also extends to seeking assistance for various illnesses (5). In China, researchers have relatively recently begun studying this field, so they have not yet established a systematic theoretical framework. Most scholars define this concept based on its literal meaning or their research needs. Xie and Xu proposes that “medical-seeking behavior refers to the process by which patients, based on information they have obtained, identify specific physical symptoms, determine the severity of their condition, and decide how to treat it” (6). According to the definition by Zhang et al., medical seeking behavior refers to the concepts, expressions, and actions taken by residents to seek medical help when they feel unwell or experience symptoms of a disease, or even when they do not feel unwell at present but feel that there is a potential risk of illness (7). The medical-seeking behavior proposed by Yao Zhaoyu’s research team refers to “the act of seeking help when people feel unwell or have some illness,” including choosing medical institutions, medical personnel, medications, and treatment methods to achieve the expected goals and results of seeking medical care and seeing a doctor. However, the research population did not take into account people who were ill but did not seek medical treatment (8). Zheng breaks down medical care into specific categories: disease management methods, choice of medical institutions, and choice of medical care methods (9). In summary, the article defines medical treatment behavior as follows: when people want to prevent disease or are already feeling unwell, they take into account subjective and objective factors and seek and use medical resources for treatment as a psychological or social behavior.

Most scholars define health care accessibility in terms of spatial accessibility, temporal accessibility, and economic accessibility. Spatial accessibility mainly refers to the distance to the nearest hospital. Temporal accessibility refers to the time spent at the nearest hospital, including the waiting time. Economic accessibility encompasses factors such as the primary payer of medical expenses, reimbursement costs, and household income. In addition, some scholars have also generalized healthcare accessibility from both supply and demand perspectives. Supply-side accessibility refers to the distance to the medical institutions, consultation time, contents of the consultation, doctor’s attitude, etc., while demand-side accessibility refers to the individual’s income, disease condition, self-assessed health condition, etc. (10). In this paper, two indicators, the time required for residents to go to the nearest medical institutions and the length of consultation, are selected to analyze the residents’ medical care behavior.

The Anderson’s initial model, formally known as the “Health Care Utilization Model,” posits that individual health care utilization is influenced by three factors: propensity characteristics, capabilities and resources, and demand factors (11). Subsequently, scholars in the field of health services management gradually refined the model to include environmental factors. It is now widely recognized in the fields of medical sociology and health services research as the mainstream model applicable to health care services research (12, 13).

## 3 Sampling methods and their limitations

### 3.1 Sampling methods

This survey was conducted under the theme of “Research on the Medical Treatment Behavior of Residents in Yicheng, Zhumadian City.” This study used the random sampling method, and based on the map of population density in Yicheng District, three public places, Tianzhong Plaza, Aijia Plaza, and Dashang New Mart, which are the top three places in terms of foot traffic, were selected as sampling locations. The questionnaire was given to participants at random during their normal routines in the morning, midday, and evening. 225 of the 230 questionnaires that were sent out were returned, resulting in a 97.83% response rate. 216 of these were deemed valid. This data does not contain any data which can identify individual. Therefore, ethical approval is exempted. Additionally, as this research is based on routinely collected data, it is exempt from informed consent requirements.

### 3.2 Research content

This study adopts the classic Anderson model for healthcare utilization in the field of healthcare demand, designing and analyzing the empirical process around the research subjects and themes. The research variables are categorized into predisposing characteristics, enabling resources, and healthcare needs, explaining residents’ healthcare utilization behavior from these three dimensions (14). Existing research has demonstrated that the Anderson model applies to China’s social context (15). The questionnaire content includes three aspects: the first part covers residents’ basic information, including gender, age group, educational attainment, marital status, occupation, monthly income, health insurance, self-assessed health status, and chronic disease prevalence. The second part addresses healthcare service accessibility, including the time required to reach the nearest healthcare facility and the duration of the most recent visit to that facility. The third part concerns healthcare-seeking behavior, including how individuals recover their health after falling ill, how they choose healthcare facilities for minor illnesses and the reasons behind their choices, how they choose healthcare facilities for major illnesses and the reasons behind their choices, whether they have had experience with primary care facilities, and their suggestions for primary care facilities. Descriptive statistics were conducted on each section to understand the healthcare-seeking behavior of residents in Yicheng District, Zhumadian City, as well as the factors influencing residents’ different healthcare-seeking behaviors.

### 3.3 Research methods

Based on this, a database was established using EpiData3.1, and the data were processed using SPSS 26.0. The variables of the three dimensions were then analyzed in interaction with the tiered diagnosis and treatment model. In subsequent multiple logistic regression analyses, we used the multiple independent samples rank sum test and multiple comparison analysis to examine whether there were significant differences in residents’ preferred healthcare institutions for common illnesses and major illnesses based on individual characteristics and healthcare service accessibility. This allowed us to identify factors influencing residents’ healthcare choice preferences and propose recommendations for rational healthcare utilization. Count data were analyzed using the chi-square test, with *p* < 0.05 considered statistically significant.

### 3.4 Sample limitations

The sample’s age and occupational structure differed from the overall population demographics; in particular, the percentage of individuals over 50 is less than 5%, which may lead to an underestimation of the demand for hierarchical treatment for older individuals.

Due to the presence of highly educated individuals in the sampling locations within commercial areas, both students and these highly educated groups were overrepresented. In the future, the study will employ stratified sampling and supplement community sampling sites with a “15-min medical circle” coverage.

In the section of the study on arrival time to healthcare facilities, the fact that people with different modes of travel have different arrival times to the same hospital from the same location was not taken into account, as the mode of transportation was not explicitly studied. In the future, the method of measuring travel time and other influencing factors will be broken down. Previous studies have shown that stratified sampling can more comprehensively explore the factors that influence patients’ intentions to seek medical treatment for common diseases (16, 17).

## 4 Current situation of medical behavior of residents in Yicheng District

### 4.1 Basic information about the population

Table 1 shows the basic profile of the residents. Of the 216 valid samples surveyed, 99 were male and 117 were female, for 45.83 and 54.17%, respectively, suggesting a generally balanced male-to-female ratio. The majority, 104 people, are within the 18 to 29 age bracket, accounting for 48.15% of the total people, followed by 55 people aged 30 to 39, constituting 25.46% of the total. Over half of the sample, totaling 133 people, possessed a bachelor’s degree or above, constituting 61.57% of the total; in contrast, 31 people had an education level of junior high school or below, representing 14.35% of the total. Of the respondents, 83 were students, for 38.43% of the total; whereas, farmers, workers, and other occupations had the least representation, with merely 9 respondents, constituting 4.17% of the total. Regarding health insurance composition, 63 individuals possess basic health insurance for urban workers, for 29.17% of the total; 132 people have basic health insurance for urban and rural residents, accounting for 61.11% of the total. A large number of respondents rated their health state as excellent, including 104 people, or 48.15% of the total, followed by 51 respondents who rated their health as very satisfactory, accounting for 23.61% of the total. Of the respondents, 42 had chronic diseases, for 19.44%, and 174 had none, totaling 80.56% of the total respondents.

**Table 1:** Individual characteristics of the population.

Variable		Number of people	Percentage composition (%)
Gender	Male	99	45.83
	Female	117	54.17
Age (years)	<18	2	0.93
	18 ~ 29	104	48.15
	30 ~ 39	55	25.46
	40 ~ 49	48	22.22
	50 ~ 59	4	1.85
	≥60	3	1.39
Education level	Below junior high school	31	14.35
	High school/middle school/technical school	29	13.43
	Bachelor’s degree/college	133	61.57
	Master’s degree and above	23	10.65
Marital status	Unmarried	114	52.78
	Married	96	44.44
	Divorcee	4	1.85
	Widowed	2	0.93
Occupation	Peasants	9	4.17
	Workers	9	4.17
	A private firm	38	17.59
	Staff of state-owned enterprises and institutions	68	31.48
	Schoolchildren	83	38.43
	Else	9	4.17
Average monthly income	<2000	61	28.24
	2,000 ~ 4,000	50	23.15
	4,000 ~ 6,000	46	21.3
	6,000 ~ 8,000	35	16.2
	≥8,000	24	11.11
Medical insurance type	Basic medical insurance for urban workers	63	29.17
	Basic medical insurance for urban and rural Residents	132	61.11
	Commercial medical insurance	13	6.02
	No health insurance, out of pocket	8	3.7
Satisfaction with physical condition	Good	51	23.61
	Better	104	48.15
	Average	50	23.15
	Poor	11	5.09
	Very poor	0	0
History of chronic diseases	Yes	42	19.44
	No	174	80.56

### 4.2 Accessibility of health services

Among the survey respondents, 77 people, or 35.65% of the total, reach the nearest medical institutions in less than 15 min, while 103 people, or 47.69%, take 15 to 30 min. The length of consultation refers to the total time used on examination, diagnosis, and treatment of the illness. The largest number of people, 75, or 34.72% of the total, were consulted for a duration of 15 to 30 min at their most recent visit to a healthcare facility, followed by 69 people, or 31.94%, who had a consultation time of 30 to 60 min (see Table 2).

**Table 2:** Accessibility of health services.

Variable		Number of people	Percentage composition (%)
Time to the nearest medical facility	<15 min	77	35.65
	15 ~ 30 min	103	47.69
	30 ~ 45 min	27	12.5
	> 45 min	9	4.17
Length of consultation at last visit to a health-care facility	<15 min	28	12.96
	15 ~ 30 min	75	34.72
	30 ~ 60 min	69	31.94
	1 ~ 2 h	39	18.06
	>2 h	5	2.31

### 4.3 Residents’ health care behavior

Of the 216 survey respondents, 128, or 59.26% of the total, would choose to go to a medical facility after becoming ill; 78, or 36.11% of the total, would choose to self-medicate; and 10, or 4.63%, would choose to let it run its course and recover.

Seventy-six residents preferred primary medical care after suffering from common and frequent illnesses, accounting for only 35.19% of the total; 111 preferred hospitals, accounting for 51.39% of the total. Most residents preferred tertiary hospitals after suffering from major diseases, accounting for 66.67% of the total number of people surveyed, followed by 65 people choosing secondary hospitals, accounting for 30.09% of the total number of people surveyed (see Table 3).

**Table 3:** Residents’ choice of healthcare facilities after illness.

Variable		Number of people	Percentage composition (%)
Preferred healthcare provider for common illnesses and diseases	Primary medical institutions	76	35.19
	Level II hospitals	58	26.85
	Tertiary hospitals	53	24.54
	Individual practice	26	12.04
	Else	3	1.39
Preferred healthcare provider for major illnesses and diseases	Primary medical institutions	4	1.85
	Level II hospitals	65	30.09
	Tertiary hospitals	144	66.67
	Individual practice	3	1.39
	Else	0	0

Among the sample of this survey, 115 people, or 53.24% of the total, had the experience of primary care; 101 people, or 46.76% of the total, did not have the experience of primary care. Of the 216 residents surveyed, 171, or 79.17% of the total, suggested improving the medical standards of healthcare personnel, followed by 158, or 73.15%, who suggested bringing in professionals to strengthen the construction of the medical team (see Table 4).

**Table 4:** Residents’ experience of primary care and suggestions for primary medical institutions.

Variable		Number of people	Percentage composition (%)
History of chronic diseases	Yes	115	53.24%
	No	101	46.76%
Suggestions for primary medical institutions	Improvement of the standard of care of health-care personnel	171	79.17%
	Introducing professionals and strengthening the medical team	158	73.15%
	Reduced prices of medical services or pharmaceuticals	105	48.61%
	Increase in health insurance reimbursement rates	87	40.28%
	Improvement of service attitudes of health care workers	76	35.19%
	Configuration of sophisticated medical equipment	128	59.26%
	Improvement of the medical environment	85	39.35%

## 5 Differential analysis of medical behavior of residents in Yicheng District

The paper focuses on the differences in residents’ health care behaviors and organizes the variables according to the order of the questionnaire options in natural number coding. The study starts at 1, with multiple-choice options assigned as 0 and 1, where 1 indicates selection and 0 indicates non-selection. The dependent variable involves the choice of health restoration, the preferred healthcare institution for common diseases (multimorbidities), and the preferred healthcare institution for major illnesses. The multi-sample rank-sum test and multiple comparisons were conducted using the health recovery method, preferred medical institutions for common diseases (multimorbidity), and preferred medical institutions for major diseases as dependent variables, while individual sample characteristics, accessibility of medical and health services, and reasons for selecting medical institutions served as independent variables.

### 5.1 Differential analysis of the medical behavior of residents with different characteristics

#### 5.1.1 Differential analysis of residents with different characteristics in choosing health recovery methods

Multi-sample rank-sum test was conducted on residents’ choice of health recovery after illness with different individual characteristics, and the results are shown in Table 5: residents’ age group (H = 14.787, *p* = 0.011), education level (H = 25.412, *p* < 0.001), occupation (H = 11.231, *p* = 0.047), self-assessed health status (H = 11.786, *p* = 0.008), and the presence of chronic diseases (H = 5.151, *p* = 0.023) were statistically tested to show statistically significant differences, while gender (H = 0.550, *p* = 0.458), marital status (H = 7.523, *p* = 0.057), average monthly income (H = 6.725, *p* = 0.151), and the type of health insurance coverage enrollment (H = 2.925 *p* = 0.403) The differences were not statistically significant after testing.

**Table 5:** Comparison of health recovery options for residents with different individual characteristics.

Variable		Number of people	Kruskal-Wallis H	*P*-value
Gender	Male	99	0.550	0.458
	Female	117		
Age (years)	<18	2	14.787	0.011
	18 ~ 29	104		
	30 ~ 39	55		
	40 ~ 49	48		
	50 ~ 59	4		
	≥60	3		
Education level	Below junior high school	31	25.412	<0.001
	High school/middle school/technical school	29		
	Bachelor’s Degree/college	133		
	Master’s degree and above	23		
Marital status	Unmarried	114	7.523	0.057
	Married	96		
	Divorcee	4		
	Widowed	2		
Occupation	Peasants	9	11.231	0.047
	Workers	9		
	A private firm	38		
	Staff of state-owned Enterprises and Institutions	68		
	Schoolchildren	83		
	Else	9		
Average monthly income	<2000	61	6.725	0.151
	2000 ~ 4,000	50		
	4,000 ~ 6,000	46		
	6,000 ~ 8,000	35		
	≥8,000	24		
Medical insurance type	Basic medical insurance for urban workers	63	2.925	0.403
	Basic medical insurance for urban and rural residents	132		
	Commercial medical insurance	13		
	No health insurance, out of pocket	8		
Satisfaction with physical condition	Good	51	11.786	0.008
	Better	104		
	Average	50		
	Poor	11		
	Very poor	0		
History of chronic diseases	Yes	42	5.151	0.023
	No	174		

Multiple comparisons between several groups were made for the results of the rank sum test for statistical significance (see Table 6), more residents in the age group of 18–29 years old chose to go to a medical institutions for their health recovery modality than those in the age group of 50–59 years old, and the difference was statistically significant (*p* = 0.044), there was a statistically significant difference in the choices of the health recovery modality of the residents whose education level was bachelor’s degree (junior college) versus those who were junior high school and below (*p* = 0.001), there is a significant difference in the choice of health recovery methods between residents with education level of bachelor’s degree (junior college) and master’s degree and above (*p* < 0.001), and bachelor’s degree (junior college) students are more inclined to choose to go to medical institutions; there is a significant difference in the choice of health recovery methods between residents with better and poorer self-assessment of their health (*p* = 0.032), and residents who consider their health better are more likely to choose to go to medical institutions for treatment after they fall ill were more likely to choose to go to a medical institutions after an illness.

**Table 6:** A two-by-two comparison of health recovery choices of residents with different individual characteristics.

Variable	Sample 1-sample 2	Adj. significance
Age groups	18 ~ 29 years old −50 ~ 59 years old	0.044
Educational attainment	Bachelor’s degree/college-below junior high school	0.001
	Bachelor’s degree/college-master’s degree and above	<0.001
Self-assessed health status	Better-poor	0.032

#### 5.1.2 Differential analysis of preferred medical institutions for residents with different characteristics

As shown in Table 7, there is a statistically significant difference in the preferred medical institutions after the experience of common diseases (multiple diseases) among residents with varying educational levels (H = 10.411, *p* = 0.015) and occupations (H = 19.038, *p* = 0.002). However, no statistically significant differences were observed in the preferred medical institutions based on gender (H = 0.366, *p* = 0.545), age group (H = 7.367, *p* = 0.195), marital status (H = 4.076, *p* = 0.253), average monthly income (H = 4.906, *p* = 0.297), type of health insurance participation (H = 3.269, *p* = 0.352), self-assessed health status (H = 1.832, *p* = 0.608), or the incidence of a chronic disease (H = 0.772, *p* = 0.380) among residents suffering from common diseases (multiple diseases). No statistical differences were noticed; however, significant differences in preferred medical institutions following major diseases were noted among residents based on education levels (H = 19.203, *p* < 0.001), occupations (H = 28.946, *p* < 0.001), and self-assessed health statuses (H = 8.466, *p* = 0.037). Conversely, no significant differences were found regarding preferred medical institutions among different genders (H = 1.875, *p* = 0.171), age groups (H = 7.056, *p* = 0.217), marital status (H = 3.851, *p* = 0.278), average monthly income (H = 6.867, *p* = 0.143), type of health insurance enrollment (H = 3.738, *p* = 0.291), or the presence of chronic diseases (H = 2.741, *p* = 0.098) among residents suffering from major diseases.

**Table 7:** Comparison of preferred healthcare facilities after illness among residents with different individual characteristics.

Variable		Number of people	Preferred Healthcare provider for common illnesses and diseases		Preferred healthcare provider for major illnesses and diseases	
			Kruskal-Wallis H	*p*-value	Kruskal-Wallis H	*P*-value
Gender	Male	99	0.366	0.545	1.875	0.171
	Female	117				
Age (years)	<18	2	7.367	0.195	7.056	0.217
	18 ~ 29	104				
	30 ~ 39	55				
	40 ~ 49	48				
	50 ~ 59	4				
	≥60	3				
Education level	Below junior high school	31	10.411	0.015	19.203	<0.001
	High school/middle school/technical school	29				
	Bachelor’s degree/college	133				
	Master’s degree and above	23				
Marital status	Unmarried	114	4.076	0.253	3.851	0.278
	Married	96				
	Divorcee	4				
	Widowed	2				
Occupation	Peasants	9	19.038	0.002	28.946	<0.001
	Workers	9				
	Private firm	38				
	Staff of state-owned enterprises and institutions	68				
	Schoolchildren	83				
	Else	9				
Average monthly income	<2000	61	4.906	0.297	6.867	0.143
	2000 ~ 4,000	50				
	4,000 ~ 6,000	46				
	6,000 ~ 8,000	35				
	≥8,000	24				
Medical insurance type	Basic medical insurance for urban workers	63	3.269	0.352	3.738	0.291
	Basic medical insurance for urban and rural residents	132				
	Commercial medical insurance	13				
	No health insurance, out of pocket	8				
Satisfaction with physical condition	Good	51	1.832	0.608	8.466	0.037
	Better	104				
	Average	50				
	Poor	11				
	Very poor	0				
History of chronic diseases	Yes	42	0.772	0.380	2.741	0.098
	No	174				

Multiple comparisons of statistically significant results from the rank-sum test are presented in Table 8. Significant differences were observed in the preferred medical institutions choice following common illness (frequent diseases) between residents with education levels of junior high school or below and those with bachelor’s degrees (junior college) (*p* = 0.028), and between residents with education levels of junior high school or below and those with master’s degrees or above (*p* = 0.025). Significant differences were also found in the preferred medical institutions choice following common illness (frequent diseases) between residents employed as workers and self-employed individuals (*p* = 0.031), between workers and students (*p* = 0.006), and between workers and employees of government, state-owned enterprises, and public institutions (*p* = 0.002); there are significant differences in the preferred medical institutions chosen by residents with junior high school education or below compared to those with bachelor’s degrees (or associate degrees) after suffering from a serious illness (*p* = 0.003). Similarly, there are significant differences between residents with junior high school education or below and those with master’s degrees or higher (*p* = 0.001). Furthermore, there are significant differences in the preferred medical institutions chosen by residents who are workers compared to self-employed individuals after suffering from a serious illness (*p* = 0.006). Significant differences also exist between workers and students (*p* < 0.001), and between workers and employees of government, state-owned enterprises, and public institutions (p < 0.001). Residents with lower educational attainment and those employed as workers are more likely to seek treatment at lower-level medical institutions after falling ill.

**Table 8:** A two-by-two comparison of preferred healthcare facilities after illness among residents with different individual characteristics.

Variable	Preferred healthcare provider for common illnesses and diseases		Preferred healthcare provider for major illnesses and diseases	
	Sample 1-sample 2	Adj. significance	Sample 1-sample 2	Adj. significance
Education level	Below junior high school-bachelor’s Degree/College	0.028	Below junior high school-bachelor’s degree/college	0.003
	Below junior high school-master’s degree and above	0.025	Below junior high school-master’s degree and above	0.001
Occupation	Workers-a private firm	0.031	Workers-a private firm	0.006
	Workers-Schoolchildren	0.006	Workers-schoolchildren	<0.001
	Workers-staff of state-owned enterprises and institutions	0.002	Workers-staff of state-owned enterprises and institutions	<0.001

### 5.2 Differential analysis of the accessibility of health services

As shown in Table 9, the difference in the length of consultation of the most recent visit to the healthcare facility (H = 14.379, *p* = 0.006) was statistically significant for the difference in the choice of healthcare facility after the resident suffered from a common disease (multiple illnesses), and the difference in the time it took the resident to get to the nearest healthcare facility (H = 5.118, *p* = 0.163) was statistically significant for the difference in the choice of healthcare facility after the resident suffered from a common disease (multiple illnesses) significance; the difference in the length of consultation and treatment at the nearest medical institutions (H = 9.587, *p* = 0.048) was statistically significant for the difference in residents’ choice of medical institutions after suffering from a major illness, and the difference in the time it took residents to get to the nearest medical institutions (H = 2.604, *p* = 0.457) was statistically significant for the difference in residents’ choice of medical institutions after suffering from a major illness.

**Table 9:** Comparison of different health care service accessibility on residents’ choice of health care facilities after falling ill.

Variable		Number of people	Preferred healthcare provider for Common illnesses and diseases		Preferred healthcare provider for major Illnesses and diseases	
			Kruskal-Wallis H	*P*-value	Kruskal-Wallis H	*P*-value
Time to the nearest medical facility	<15 min	77	5.118	0.163	2.604	0.457
	15 ~ 30 min	103				
	30 ~ 45 min	27				
	> 45 min	9				
Length of consultation at last visit to a health-care facility	<15 min	28	14.379	0.006	9.587	0.048
	15 ~ 30 min	75				
	30 ~ 60 min	69				
	1 ~ 2 h	39				
	>2 h	5				

Multiple comparisons were performed on statistically significant results from the rank-sum test. Results with adjusted *p*-values less than 0.05 are presented in Table 10. Significant differences were observed in the selection of healthcare facilities by residents after contracting common diseases (frequent diseases) between the 30–60 min and over 2-h consultation duration groups (*p* = 0.014), and between the 1–2 h and over 2-h consultation duration groups (*p* = 0.028). Additionally, a significant difference was found in the selection of healthcare facilities by residents after contracting serious diseases between the 30–60 min and under 15-min consultation duration groups (*p* = 0.047).

**Table 10:** A two-by-two comparison of different health care accessibility on residents’ choice of health care provider after illness.

Variable	Preferred healthcare provider for common illnesses and diseases		Preferred healthcare provider for major illnesses and diseases	
	Sample 1-sample 2	Adj. significance	Sample 1-sample 2	Adj. significance
Duration of treatment	30 ~ 60 min-more than 2 h	0.014	1 ~ 2 h-15 min or less	0.047
	1 ~ 2 h-more than 2 h	0.028		

### 5.3 Differential analysis of residents’ preferred medical institutions for illnesses

Table 11 shows that residents with common diseases prefer medical institutions for reasons such as good medical technology (H = 16.525, *p* < 0.001), reasonable medical costs (H = 12.053, *p* = 0.001), high reimbursement ratio of health insurance (H = 11.573, *p* = 0.001), good medical equipment (H = 16.905, *p* < 0.001), good medical environment (H = 20.952, *p* < 0.001), and acquaintances (H = 4.772, *p* = 0.029). After a statistical test showed that the difference was statistically significant, the health insurance reimbursement ratio is more willing to choose primary health care institutions, while residents preferred the hospital because of the hospital’s good level of medical technology, medical equipment, and environment, as well as the fact that the hospital has acquaintances. A favorable service attitude (H = 1.943, *p* = 0.163), proximity to medical care, convenience of access to medical care (H = 2.954, *p* = 0.086), and recommendations from family members or other relatives and friends (H < 0.001, *p* = 0.983) were examined, but there were no statistically significant differences.

**Table 11:** Comparison of residents’ preferred medical institutions for common diseases.

Variable	Number of people	Kruskal-Wallis H	*P*-value
Good medical technology	106	16.525	<0.001
Reasonable medical expenses	117	12.053	0.001
High rate of reimbursement by health insurance	79	11.573	0.001
Good service attitude	65	1.943	0.163
Good medical equipment	69	16.905	<0.001
Good medical environment	61	20.952	<0.001
Close proximity and easy access to medical care	139	2.954	0.086
Have an acquaintance at the hospital	36	4.772	0.029
Family or other relatives or friends suggest	31	<0.001	0.983

According to Table 12, among the reasons why residents preferred medical institutions after suffering from major illnesses were the reasonable medical costs (H = 5.822, *p* = 0.016), the high reimbursement ratio of medical insurance (H = 4.899, *p* = 0.027), the favorable service attitude (H = 9.713, *p* = 0.002), and the presence of acquaintances (H = 5.442, *p* = 0.020). After a statistical test showed the difference residents choose different levels of hospitals for different statistically significant reasons. For example, citizens who are concerned about the cost of medical care are more likely to pick second-tier hospitals after a serious illness. Good medical technology level (H = 1.673, *p* = 0.196), good medical equipment (H = 2.168, *p* = 0.141), good medical environment (H = 2.395, *p* = 0.122), proximity to medical treatment, convenient to consult (H = 0.312, *p* = 0.576), and family or other relatives and friends suggested (H = 1.728, *p* = 0.189) after testing. The difference was not statistically significant.

**Table 12:** Comparison of residents’ preferred medical institutions for major illnesses.

Variable	Number of people	Kruskal-Wallis H	*P*-value
Good medical technology	190	1.673	0.196
Reasonable medical expenses	76	5.822	0.016
High rate of reimbursement by health insurance	82	4.899	0.027
Good service attitude	54	9.713	0.002
Good medical equipment	168	2.168	0.141
Good medical environment	128	2.395	0.122
Close proximity and easy access to medical care	56	0.312	0.576
Have an acquaintance at the hospital	37	5.442	0.020
Family or other relatives or friends suggest	39	1.728	0.189

## 6 Results and discussion

### 6.1 The phenomenon of residents’ access to medical care being higher rather than lower and more familiar than less familiar

Only 35.19% of the residents preferred primary healthcare organizations to seek medical treatment after suffering from common diseases, and 53.24% had the experience of primary first medical treatment, which indicates that there is still the phenomenon of choosing a healthcare institution from a higher rather than a lower level healthcare organization among the residents. The results of the study based on Anderson’s health service utilization model show that tendency and need factors have a more significant impact on residents’ healthcare behavior (18), and the traditional concept of “delaying minor illnesses and resisting major illnesses” affects their choice of healthcare (19), with residents with a lower level of education and those who are employed as workers being more inclined to choose primary medical institutions, and more likely to choose secondary hospitals when suffering from a major illness. When suffering from serious illnesses, they also choose secondary hospitals more often, i.e., the higher the level of education, the higher the level of medical institutions they tend to choose, while residents who work as farmers and laborers tend to choose lower-level medical institutions (20). In Li et al.’s article, it was also noted that when a major illness struck, people with better self-assessed health status were more inclined to choose tertiary hospitals than those with poor self-assessed health status. That’s given that individuals with poor self-perceived health status choose proximity or accessibility when selecting hospitals, as people with self-perceived health status choose quality of care (21).

The results of the survey show that there is also a phenomenon of residents seeking medical treatment based on familiarity rather than alienation, with many residents choosing to contact their acquaintances after falling ill to inquire about beds, doctors, medications, and methods of treatment, and that medical institutions “where there are acquaintances” are more likely to be the first choice of medical institutions for them. The advice of family members or other relatives and friends is also a factor influencing residents’ choice of medical care, and the more supportive a significant other is of a resident’s choice of medical care, the greater the likelihood that the resident will take action.

### 6.2 Inadequate service capacity of primary medical institutions

The implementation of the hierarchical diagnosis and treatment system still needs to be promoted more aggressively because some residents value the level of medical technology, medical equipment, and the medical environment of medical institutions more than others, and primary health care institutions are unable to meet the residents’ requirements in these aspects. According to this survey, residents prefer going to hospitals when choosing medical institutions over primary medical institutions. Residents have chosen primary medical institutions due to their reasonable costs and high medical insurance reimbursement. According to the survey, the most typical suggestions for primary healthcare organizations were to improve the medical standards of the staff, hire professionals, and provide them with sophisticated medical equipment. That shows that primary healthcare organizations urgently need to improve on two aspects: talent and equipment. Medical equipment is another factor that results in residents being unwilling to choose primary healthcare organizations for medical treatment. The precision of the medical equipment in primary healthcare organizations, which may have been purchased many years ago, inevitably has many shortcomings compared to the equipment in big hospitals. Healthcare talents are the foundation of healthcare services, and many healthcare talents are unwilling to work in primary healthcare organizations due to the low salary and poor career development opportunities of primary healthcare organizations. Primary healthcare organizations’ medical equipment is inevitably less sophisticated when compared to that of large hospitals.

The accessibility of medical institutions is one of the key factors influencing residents’ choice of medical institutions, which is specifically manifested in the fact that the better the accessibility of a medical institution, the greater the likelihood of residents choosing that medical institution (22). The survey shows that 35.65% of the total number of residents spend less than 15 min to get to the nearest medical institution, with most of them concentrating on 15–30 min, which indicates that the accessibility and service capacity of medical institutions still need to be further improved, and that we need to give play to the important role of primary medical institutions in the construction of the “15-min healthcare circle” to realize the principle of “resolving minor illnesses in the community” and to achieve the goal of “resolving minor illnesses in the community.” It is necessary to play an important role in the construction of the “15-min medical circle,” to realize the goal of “resolving minor illnesses in the community and treating major illnesses in hospitals”.

## 7 Recommendations for countermeasures

### 7.1 Enhancing residents’ medical health literacy and guiding them to seek medical care in an orderly manner

The survey results show that awareness of the tiered healthcare system has a certain impact on residents’ choice of primary care provider, which is consistent with the findings of Liu et al. (23). And the basis for residents’ choice of medical treatment is their level of medical health literacy. The phenomenon of passive medical treatment, self-treatment, and blindly choosing large hospitals for medical treatment is caused by a lack of basic medical knowledge, whereas the development of medical health literacy will help the residents in assessing the severity of their disease conditions and in making scientific decisions concerning the treatment method and the medical institution. Zhumadian City in recent years has closely followed the popularization of health knowledge, rational diet, fitness, and 15 other special actions to carry out health promotion work, and through the prescription of exercise as a cure for prevention, the level of residents’ health literacy has been improved, but the popularization of medical knowledge should continue to be promoted so that they set up a scientific concept of medical care and make reasonable choices of medical institutions. We should actively use community bulletin boards to fully leverage the power of public media. Furthermore, schools and communities should hold lectures on the basic concepts of medicine and widely distribute knowledge about disease prevention and treatment and ways to help residents better comprehend and accept primary health care institutions and hierarchical diagnostics and treatment. Residents also should be influenced to change their views about medical care and their patterns of behavior, choosing nearby primary medical and health care institutions for medical treatment.

Graded diagnosis and treatment efficiently address the issue of difficulty obtaining doctors; nonetheless, the current rate of residents visiting primary medical institutions remains low due to insufficient exposure to this system, as well as a lack of awareness and acceptance by residents. Furthermore, many residents are still concerned that obtaining primary care may delay their therapy. Many residents have yet to abandon their rejection of primary care, fearing that obtaining care at the main level may prolong their disease. Medical institutions at all levels should actively publicize the tiered diagnosis and treatment system through lectures, in-hospital bulletin boards, posters, banners, and other means to educate residents about the convenience of primary care and the benefits of medical insurance reimbursement. In addition, we should use newspapers, radio, television, microblogs, WeChat, and other media to widely and comprehensively carry out publicity and education on knowledge related to hierarchical diagnosis and treatment for groups of different ages and educational levels to increase residents’ awareness and acceptance of hierarchical diagnosis and treatment so that they can understand the meaning of hierarchical diagnosis and treatment, change their concepts of medical care, and actively choose primary care as their first point of contact.

### 7.2 Enhancement of primary health care services

The graduated diagnosis and treatment system is an important part of the medical system reform. The construction of the graduated diagnosis and treatment system is the fundamental strategy to reconstruct China’s medical and health care service system and improve the efficiency of the service (24), and the primary first diagnosis and treatment system is the important foundation of the graduated diagnosis and treatment system, and comprehensively improve the service capacity of primary medical institutions is an important part of the construction of the graduated diagnosis and treatment system. The service capacity of primary medical institutions largely depends on the performance of their talent teams; however, the current shortage of skilled personnel and the low technical proficiency within these institutions are significant factors contributing to residents’ reluctance to seek treatment there. Zhumadian City is now constantly innovating the recruitment mode, optimizing the recruitment process, optimizing the salary structure, improving the treatment of personnel, and opening up a green channel for talents. The healthcare talent has increased. Talent team building is a continuous process. To fully implement the Henan Province “369” talent project, it is essential to utilize the resources of Zhumadian City, including the three hospitals, the general practitioner training base, and the Zhumadian School of Health. This should be based on grassroots doctors and involve establishing specialized training channels for grassroots health personnel in Zhumadian City. Additionally, large hospitals should include medical personnel in their assessments for grassroots support services and provide better promotion opportunities for those with several years of experience in grassroots service.

One of the main reasons hindering the development of primary medical institutions is the irrational allocation of medical resources and the inability to effectively meet the population’s medical needs. This requires the government to continue to provide strong support to grassroots medical institutions while taking various measures to attract talent and strengthen the training of existing medical personnel. To motivate and encourage grassroots medical personnel to take initiative in their work, it is also necessary to improve their salary levels and establish a robust performance appraisal and incentive mechanism. At the same time, it is necessary to consider the convenience of medical care from the perspective of residents and reasonably increase the number of grassroots medical institutions in accordance with local conditions. Standardized construction of 143 township health centers across the city will be carried out, and 962 village health rooms that do not meet the standards will be newly built, expanded, or renovated in batches. In addition, by conducting performance evaluations of medical staff at large hospitals and establishing a comprehensive and complementary medical and health service compensation incentive mechanism, expert physicians who regularly provide assistance and consultation services at grassroots medical institutions will be included in the evaluation scope, thereby achieving effective coordination of medical and health resources between large hospitals and grassroots medical institutions (25). Finally, with the advent of the information age, leveraging the internet for remote network-based medical case analysis and providing diagnostic and treatment knowledge education to primary care medical staff can facilitate the downward flow of high-quality resources from large hospitals to primary care facilities (26).

### 7.3 Improvement of the mechanism for guaranteeing access to health care at the grass-roots level

Medical institutions at all levels should clarify their position in the health care service system and play their respective roles. The diagnosis and treatment of critical illnesses and difficult diseases are the main focus of tertiary hospitals. In addition to being responsible for providing technical guidance and training to primary healthcare organizations, county hospitals are also responsible for the diagnosis and treatment of critically ill patients, as well as for the treatment of generally difficult diseases. It also accepts patients who are in recovery after being referred by tertiary hospitals and is responsible for referring patients with difficult and complex diseases upward. Primary healthcare facilities should take advantage of their close connections with residents with the aim of thoroughly understanding their illnesses, providing them with the medicines they need, treating common and frequent diseases, and offering high-quality, reasonably priced medical services.

The current differences in reimbursement rates for various levels of medical institutions do not yet offer an effective incentive for participants to give preference to primary health care institutions. So the reimbursement rate gap between primary health care institutions and other health care institutions should be widened, and the proportion of reimbursement rates for visits to primary health care institutions should be appropriately increased. The use of the economic leverage of health insurance reimbursement and the adoption of differentiated reimbursement standards for different levels of medical institutions can standardize residents’ choice of medical institutions. On this basis, to promote an uninterrupted patient flow, the reimbursement rate should be increased for those who receive a first consultation and referral at the primary level and reduced for those who do not follow the prescribed procedures.

## Data Availability

The raw data supporting the conclusions of this article will be made available by the authors, without undue reservation.
