# Supplementary material for: Study on the medical behaviors of residents in Yicheng District of Zhumadian
Source: Front Public Health. 2026 Jan 6;13:1610825. doi: 10.3389/fpubh.2025.1610825 (PMC12816218; doi:10.3389/fpubh.2025.1610825)
Supplement: Supplementary file 1 [file Data_Sheet_1.docx]

**Zhumadian Yicheng District Resident Medical Care Behavior Survey Questionnaire**

1. What is your gender?

☐ Male ☐ Female

2. What is your age?

☐ Under 18 ☐ 18–29 ☐ 30–39 ☐ 40–49 ☐ 50–59 ☐ 60 or older

3. What is your educational level?

☐ Below junior high school ☐ High school/vocational school/technical school ☐ Bachelor's degree/associate degree ☐ Master's degree or above

4. What is your marital status?

☐ Unmarried ☐ Married ☐ Divorced ☐ Widowed

5. What is your occupation?

☐ Farmer ☐ Worker ☐ Self-employed individual ☐Government/state-owned enterprise/public institution employee ☐Student ☐Other

6. What is your monthly income?

☐Below 2,000 yuan ☐2,000–4,000 yuan ☐4,000–6,000 yuan ☐6,000–8,000 yuan ☐8,000 yuan and above

7. What type of medical insurance do you have?

☐Urban employee basic medical insurance ☐Rural and urban resident basic medical insurance ☐Commercial medical insurance ☐No medical insurance, self-funded

8. How would you describe your current health condition?

☐Good ☐Fairly good ☐Average ☐Fairly poor ☐Very poor

9. Do you have any chronic illnesses?

☐Yes ☐No

10. How long does it take to reach the nearest medical facility from your residence?

☐Within 15 minutes ☐15–30 minutes ☐30–45 minutes ☐Over 45 minutes

11. How long did your last medical consultation take?

☐Within 15 minutes ☐15–30 minutes ☐30–60 minutes ☐1–2 hours ☐Over 2 hours

12. When you have a common or frequently occurring illness, which type of medical institution do you choose first?

☐Primary care medical institution ☐Secondary hospital ☐Tertiary hospital ☐Private clinics ☐Other

13. Which type of medical institution would you choose first when suffering from a serious illness?

☐Primary healthcare institutions ☐Secondary hospitals ☐Tertiary hospitals ☐Private clinics ☐Other

14. Have you ever had an experience of primary healthcare institutions as the first point of contact?

☐Yes ☐No

15. What suggestions do you have for primary healthcare institutions? (Multiple selections allowed)

☐ Improve the medical skills of healthcare personnel ☐ Introduce professional talent and strengthen medical team building ☐ Reduce medical service/medication prices ☐ Increase medical insurance reimbursement ratios ☐ Improve the service attitude of healthcare personnel ☐ Equip with advanced medical devices ☐ Improve the medical environment

16. When you fall ill in daily life, what type of hospital would you choose for treatment? (Multiple selections allowed)

☐High medical technology standards ☐Reasonable medical costs ☐High medical insurance reimbursement rates ☐Good service attitude ☐Advanced medical equipment ☐Good medical environment ☐Convenient location and easy access ☐Recommendations from family members or other relatives

17. When facing a serious illness, what type of hospital would you choose for treatment? (Multiple selections allowed)

☐High medical technology standards ☐Reasonable medical costs ☐High medical insurance reimbursement rate ☐Good service attitude ☐Advanced medical equipment ☐Good medical environment ☐Close proximity to the hospital, convenient for medical visits ☐Recommendations from family members or other relatives
